# Selection forces underlying aneuploidy patterns in cancer

**DOI:** 10.1080/23723556.2024.2369388

**Published:** 2024-06-24

**Authors:** Tamara C. Klockner, Christopher S. Campbell

**Affiliations:** a Max Perutz Labs, Vienna Biocenter Campus (VBC), Vienna, Austria; b Center for Molecular Biology, Department of Chromosome Biology, University of Vienna, Vienna, Austria; c A Doctoral School of the University of Vienna and the Medical University of Vienna, Vienna, Austria

**Keywords:** Aneuploidy patterns, cancer, selection forces, driver genes

## Abstract

Aneuploidy, the presence of an aberrant number of chromosomes, has been associated with tumorigenesis for over a century. More recently, advances in karyotyping techniques have revealed its high prevalence in cancer: About 90% of solid tumors and 50–70% of hematopoietic cancers exhibit chromosome gains or losses. When analyzed at the level of specific chromosomes, there are strong patterns that are observed in cancer karyotypes both pan-cancer and for specific cancer types. These specific aneuploidy patterns correlate strongly with outcomes for tumor initiation, progression, metastasis formation, immune evasion and resistance to therapeutic treatment. Despite their prominence, understanding the basis underlying aneuploidy patterns in cancer has been challenging. Advances in genetic engineering and bioinformatic analyses now offer insights into the genetic determinants of aneuploidy pattern selection. Overall, there is substantial evidence that expression changes of particular genes can act as the positive selective forces for adaptation through aneuploidy. Recent findings suggest that multiple genes contribute to the selection of specific aneuploid chromosomes in cancer; however, further research is necessary to identify the most impactful driver genes. Determining the genetic basis and accompanying vulnerabilities of specific aneuploidy patterns is an essential step in selectively targeting these hallmarks of tumors.

## 1. Introduction

In 1914, Theodor Boveri formulated the first hypothesis linking incorrect chromosome content with the origin of tumorigenesis based on observations made by pathologist David Paul von Hansemann at the end of the 19^th^ century.^1^ However, just how widespread aneuploidy is across many different tumor types has only been appreciated since the development of high throughput karyotyping techniques.^2–6^ Approximately 90% of solid tumors and 50–70% of hematopoietic cancers display chromosome gains and/or losses.^7^ When analyzed at the level of specific chromosomes, there are strong patterns that are observed in cancer karyotypes both pan-cancer and for specific cancer types. We use the term aneuploidy patterns to refer to copy number alterations of whole chromosomes or chromosome arms that occur at a high frequency relative to other chromosomes/arms. Strong aneuploidy patterns point toward the selection of specific aneuploid chromosomes in cancer. Such aneuploidy patterns are observed in all stages of tumorigenesis, suggesting important roles in cancer development and progression.

Although specific chromosomal arm aneuploidies rank amongst the most frequent alterations in cancer, determining the consequences of aneuploidy on cancer development has been hindered by the difficulty in engineering specific aneuploidies. Furthermore, the impact of aneuploidy on subtle expression changes of hundreds of genes simultaneously has made it difficult to determine the genetic basis behind their selection. However, recent advances in both genetic engineering of aneuploidy in human cells and bioinformatic analyses of cancer genomes have led to substantial insights into the genetic determinants of the selection of aneuploidy patterns in human cells.^6,8–13^ Determining the mechanistic basis of aneuploidy patterns will lead to a better understanding of cancer and has the potential to identify new targets for personalized cancer treatments.

In this review, we will describe the types of aneuploidy patterns observed in cancer and discuss the selection forces that potentially determine these patterns. The cellular mechanisms leading to aneuploidy formation, the general consequences of aneuploidy, and methods for engineering aneuploidy have been extensively reviewed elsewhere (compare^14–17^ and are not the focus of this review.

## 2. Types of aneuploidy patterns in cancer

Specific chromosome gains and losses are among the most common genomic alterations in cancer. For comparison, mutation of the tumor suppressor gene *TP53* is the most prevalent alteration identified within The Cancer Genome Atlas (TCGA) dataset, at 37%.^18^ The next 23 most common alterations observed in TCGA tumors are chromosome arm aneuploidies.^13^ The most common of these chromosome arm alterations include the gains of 20q (31.9%), 7p (31%), 8q (29.6%), 1q (27.8%), 7q (27.3%), and 20p (26.4%), and the loss of 17p (24.6%).^13^ These frequencies are comparable to those measured in other cancer data sets.^3,4,19,20^

**Figure 1.**
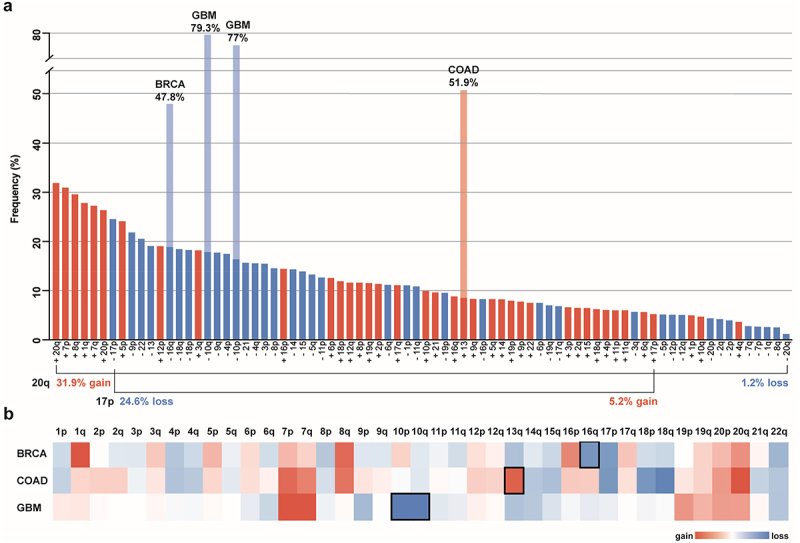
Pan-cancer and cancer type-specific aneuploidy patterns **a**. Frequency of chromosome arm aneuploidies from most to least common across all TCGA cancer types. The frequencies of cancer type-specific aneuploidy patterns highlighted in **b** are depicted in light blue and light red. The directionality of aneuploidy patterns (gain vs. loss) for chromosome arms 20q and 17p are highlighted. 20q is the most commonly gained and most infrequently lost chromosome arm, whereas 17p is the most frequently lost chromosome arm and only rarely gained. Bars for chromosome arm gains are red and losses are blue. Frequencies of chromosome arm aneuploidies are taken from reference.^13^ **b**. Heat map of the average copy number data for somatic chromosome arms of BRCA (Breast Invasive Carcinoma), COAD (Colon Adenocarcinoma), and GBM (Glioblastoma Multiforme) cancers from the TCGA data set. Copy number gains are red and copy number losses are blue. Frequencies are taken from reference.^8^ Characteristic cancer type-specific aneuploid chromosomes are surrounded by a black square.

Another important aspect of aneuploidy patterns lies in the specificity of chromosome gains vs. losses. Across cancer-types, most chromosome arms are either gained or lost, but rarely both.^3^ For instance, chromosome arm 20q, the most prevalent gained chromosome arm encompassing 31.9% of samples, is decreased in copy number in only 1.2% of tumors. Likewise, the most common chromosome arm loss, 17p, prevalent in 24.6% of TCGA tumors, is gained in a mere 5.2% of tumors,^13^ (Figure 1a). This trend toward either chromosome gain or loss suggests a strong selection bias, as any given chromosome missegregation event would create one daughter cell with an extra copy and one daughter cell lacking one copy of the chromosome. These pan-cancer aneuploidy patterns indicate that copy number changes of specific chromosomes play an important role in carcinogenesis across many different cancer types and microenvironments.^21^ However, it is not known whether particular aneuploid chromosomes are selected for in different cancer types for the same underlying reasons.

Aneuploidy patterns for individual cancer types can differ significantly from pan-cancer patterns in both the percentage and the directional bias,^3,21,22^ (Figure 1b). One example of cancer type-specific aneuploidy patterns is for chromosome 13. Although chromosome 13 is often decreased in copy number across most cancer types, colorectal and stomach cancers frequently gain chromosome 13 instead.^6,23^ For other cancer types, certain aneuploidies are considered a hallmark of the disease. The clearest example of this is in glioblastoma, where chromosome 7 gain and 10 loss are each found in ~ 70–90% of cases.^24^ Aneuploidies are also indicators of cancer subtypes. For example, chromosome 16q loss is observed in the majority of differentiated breast cancer, but only rarely in the undifferentiated form.^25^ This cancer type specificity is similar to what is observed for mutations in many oncogenes (OGs) and tumor suppressor genes (TSGs), as certain aneuploidies are observed across many different cancer types while others are more cancer-type specific.^26^ The identification of different aneuploidy patterns points toward a context-dependent selective advantage of aneuploidy-induced changes in tumor cells.

## 3. Specific aneuploidy patterns have been identified at all stages of carcinogenesis

**Figure 2.**
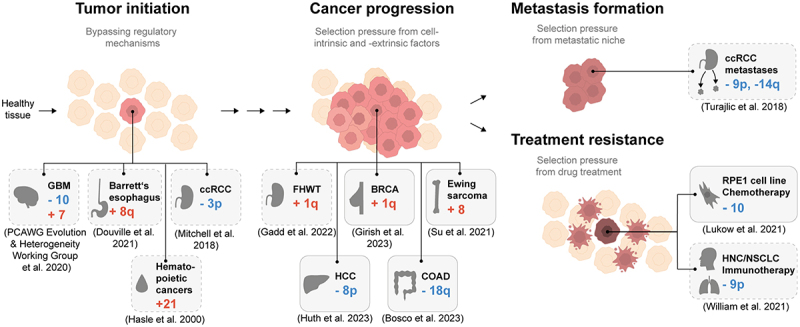
Aneuploidy patterns have been identified in all stages of cancer. Schematic of identified aneuploidy patterns in cancer or cell lines in the context of cancer evolution. The gray boxes display examples of recurring aneuploidy patterns. Boxes with dashed lines represent correlative evidence; boxes with solid lines represent direct evidence based on cell culture experiments. GBM: Glioblastoma Multiforme, ccRCC: clear cell Renal Cell Carcinoma, FHWT: Favorable Histology Wilms Tumor, BRCA: Breast Invasive Carcinoma, HCC: Hepatocellular Carcinoma, COAD: Colon Adenocarcinoma, HNC: Head and Neck Cancer, NSCLC: Non-Small Cell Lung Cancer.

Recently, progress has been made in determining the timing of mutational events in cell transformation. Top-down studies aiming to determine the evolutionary history of a cancer based on karyotype heterogeneity have concluded that aneuploidy patterns are present in all stages of oncogenesis and continue to change over time.^27,28^ These evolutionary reconstructions indicate that there are multiple roles of aneuploidies in carcinogenesis (Figure 2). In this section, we discuss aneuploidy patterns observed in tumor initiation, progression, metastasis, and therapy resistance. We will also examine the evidence for these aneuploidy patterns having a causative role in carcinogenesis. The subsequent section will elaborate on potential genetic mechanisms responsible for these frequent aneuploidies.

### 3.1. Aneuploidy patterns in tumor initiation

The presence of aneuploidy in precancerous lesions of certain cancer types raises the possibility for a causative role in tumor initiation. In a study examining tumor samples of patients with Barrett’s esophagus, investigators identified the gain of chromosome 8q in 75% of the cases (*N* = 20).^29^ Chromosome 8q gain may result from the CIN inducing stressful microenvironment attributable to acid reflux and, together with other cellular changes, contribute to cancer initiation. Abe and colleagues identified that the exposure of a Barrett’s esophagus metaplastic cell line, CP-A, to acids and deoxycholic acid resulted in the hypomethylation of α-satellite DNA. This lead to heightened expression of non-coding RNA transcribed from α-satellite sequences ultimately resulting in chromosomal instability.^30^ However, it should be noted that in their study following acid treatment 8q gain was not among the acquired aneuploidies, suggesting that the acidic conditions may be responsible for increasing the amount of aneuploidy but not the selection of 8q.

Various groups have modeled the progression of cancers over time to determine which changes to the genome act early in their evolution. Modeling of the somatic evolution of clear cell renal cell carcinoma (ccRCC) revealed that chromosome 3p is frequently lost early in oncogenesis (30–40% of 95 biopsies). Estimates date this event back as far as 30–50 years before cancer diagnosis.^31^ In another study, investigators calculated that chromosome 7 gain and 10, 17p, and 9p loss typically occur very early in the development of glioblastoma, based on the low number of single nucleotide variants (SNV) on chromosomes before amplification and an assumed mutation rate of SNV per base pair and cell division.^28^ In agreement with this, another research group comparing matched primary and recurred glioblastomas calculated that 7 gain and 9p and 10 loss are acquired 2–7 years before cancer diagnosis.^32^

Further evidence for aneuploidy playing a causative role in cancer formation comes from studies on trisomy 21. Individuals with an extra copy of chromosome 21 have a substantially increased risk of developing hematopoietic cancers, but, conversely, the risk for developing solid tumors is significantly decreased.^33,34^ Interestingly, trisomy 21 is the most frequent aneuploidy in blood cancers from euploid individuals.^35,36^ These observations together suggest that the gain of chromosome 21 contributes to the development of hematopoietic tumorigenesis.

Experiments in mice have shown that despite mostly having a negative effect on cellular fitness, aneuploidy can promote tumorigenesis. For instance, Williams and colleagues determined that the effect of aneuploidy on transformation capacity in mouse embryonic fibroblasts is specific to the identity of the aneuploid chromosome, i.e. trisomy 13 inhibiting, trisomy 19 neutral and trisomy 16 supporting transformation.^37^ Mouse models for increased chromosome missegregation through centrosome amplification, kinetochore defects, or decreased spindle assembly checkpoint activity have demonstrated an increased incidence of tumor formation, suggesting a role for either chromosome instability (CIN) or aneuploidy in this process.^38–43^ Furthermore, for certain cancer types, this resulted in strong aneuploidy patterns, suggesting that particular chromosomes are acting as cancer drivers.^38,44^ However, the causative role of individual aneuploidies as a primary source of tumor initiation is still under debate, as engineered aneuploidy typically leads to decreased proliferation, transformation, and tumor growth.^37,45,46^

### 3.2. Aneuploidy patterns in cancer progression

In patient survival analyses comparing cancer karyotypes, higher levels of aneuploidy generally correlate with poor prognosis.^47^ Particular aneuploid chromosomes are mostly associated with unfavorable outcomes (87%); however, the remaining aneuploidies (13%) are associated with increased patient survival. For example, although likely rare, glioblastomas with 10p gain have a more beneficial prognosis than glioblastomas without this specific aneuploidy.^48^ Intriguingly, certain combinations of frequent aneuploidies have also been found to correlate with patient survival, suggesting a more complex relationship between aneuploidy and cancer progression.^48,49^ For instance, the coincident loss of chromosome arms 9p and 21q in liver hepatocellular carcinoma is predictive for a decreased disease free survival.^49^ Notably, cancer karyotypes often have multiple aneuploidies, and therefore combinations of aneuploidies may have an increased potential to drive tumor progression.

Even though certain aneuploidies accumulate in cancer cells at high rates, that does not necessarily mean that they directly contribute to cancer progression. Attempts to determine if specific aneuploid chromosomes contribute to tumor formation have yielded largely negative results. Mouse xenografts of human cancer cells engineered with frequently observed aneuploidies generally do not promote tumor formation.^45–48^ Furthermore, when the tumors that do form are resequenced, the engineered aneuploidy is frequently lost, indicating that it was acting in an inhibitory manner. These results highlight the context-sensitive nature of aneuploid chromosome selection in cancer.

Although recapitulating the advantages of pan-cancer aneuploidy patterns has been challenging, there are a few cases in which a cancer type-specific advantage of specific aneuploidies could be demonstrated in cell culture. For instance, the gain of chromosome 1q is very frequently acquired in breast cancer, among other types. Using breast cancer cell lines with and without 1q gain, Girish and colleagues could show that xenograft growth and anchorage-independent growth is dependent on the 1q gain.^11^ Similarly, the gain of chromosome 8 is prevalent in multiple types of cancer. In a Ewing sarcoma cell line harboring the specific gene fusion *EWS-FLI1*, Su and colleagues determined the cancer type-specific benefit of this aneuploidy in restoring DNA replication fidelity.^50^ In another study, liver cancer cell lines engineered with monosomy for chromosome arm 8p had accelerated migratory and invasive potential.^12^ Finally, Bosco and colleagues showed that the engineered loss of chromosome 18q, which occurs frequently in colorectal cancer, reduced the sensitivity to growth-inhibition by TGF-β.^9^ These bottom-up approaches highlight the complexity of the role of aneuploidies in cancer progression.

### 3.3. Aneuploidy patterns involved in resistance to immunotherapy and chemotherapy

The aspect of tumor progression that is most highly associated with aneuploidy is immune evasion and resistance to immunotherapy.^51^ In general, highly aneuploid tumors are associated with poor prognosis after immunotherapy.^52,53^ More recently, the loss of chromosome 9p, which is common in multiple cancer types, has been linked to immune evasion and immunotherapy resistance. In HPV negative head and neck cancer (HNC) and Non-Small Cell Lung Cancer (NSCLC), the loss of chromosome arm 9p underlies a switch from an immune hot to an immune cold tumor microenvironment and serves as a predictive marker associated with limited clinical benefit from anti-PD-(L)1 therapy.^54^ Overall aneuploidy levels and specific aneuploidies both correlate with immune evasion and immunotherapy resistance, making aneuploidy a promising clinical prognostic biomarker for immunotherapy.

However, it is still unclear if the immune evasion associated with aneuploidy primarily results from the aneuploidy itself, or from chromosomal instability. Certain types of CIN can generate cytosolic DNA, which triggers interferon-STAT1/3 and TNF – NF-κB immune signaling through the cGAS/STING pathway. In advanced stages of tumorigenesis, an adaptive reconfiguration of signaling pathways downstream of STING enables tumors to avoid the pro-inflammatory effects of type I interferons, resulting in immune evasion.^55–57^

The frequency of aneuploidies have also been observed to increase following chemotherapies. For instance, in Favorable Histology Wilms Tumors (FHWT), the gain of chromosome 1q (28% of individuals) has been linked with reduced overall survival. Gadd and colleagues identified that the number of samples with gain of chromosome 1q was significantly higher (74.5%, *N* = 51) in relapse samples compared to primary samples (47%, *N* = 45), indicative of a role of 1q in chemotherapy resistance in FHWT.^58^

Recent experiments identified the acquisition of individual aneuploidies as resistance mechanisms to chemotherapeutic drugs in cell culture.^59,60^ By adapting RPE1 cells to the chemotherapeutic drug paclitaxel after a pulse of a spindle assembly checkpoint inhibitor to induce aneuploidy, Lukow and colleagues identified the loss of one copy of chromosome 10 in separately adapted populations.^60^ In a study from our lab, we demonstrated that prolonged inhibition of the spindle assembly checkpoint in multiple non-transformed and cancer cell lines resulted in the selection of distinct aneuploidy patterns that correlated with increased drug resistance.^8^ In both studies, cell lines harboring the individual frequent monosomies were sufficient for drug resistance. Collectively, there is substantial evidence from evolution experiments in cell culture and cancer karyotype analysis demonstrating that aneuploidy patterns contribute to the acquisition of resistance phenotypes in cancer cells.

### 3.4. Aneuploidy patterns in metastases

In general, metastatic tumors have a substantially higher degree of aneuploidy than primary tumors,^61,62^ with a median copy number change of ~ 5 chromosome arms compared to ~ 3.^49^ The increase in aneuploidy following metastasis also occurs for specific chromosomal aneuploidies. For primary ccRCC, 9p and 14q loss were observed in most of the analyzed metastatic sites, which indicates a selective advantage of these aneuploidies for metastasis development.^63^ In a mouse model of metastatic renal cell carcinoma with partial 9p loss, Perelli and colleagues demonstrated that this evolutionary pressure leads to recurrent copy number alterations. In particular, the loss of mouse chromosome 16q along with the initial 9p loss results in a proliferative advantage.^64^

Metastasis is one of the few areas where the engineering of specific chromosome gains has an effect on promoting tumor formation in mouse xenografts. Human colon carcinoma cells (HCT116) engineered with trisomy of chromosome 5 displayed significantly increased invasiveness in comparison to their near-diploid progenitor cells, suggesting a role for this aneuploidy in metastasis.^48^ In conclusion, aneuploidy levels increase substantially after metastasis, and there are a few examples that indicate a selective advantage of specific aneuploidy patterns for cell invasion and metastasis.

Overall, there is substantial evidence that aneuploidy patterns play multiple roles in tumor evolution. There are strong correlations between specific aneuploidies and outcomes for tumor initiation, progression, metastasis formation, immune evasion and resistance to therapeutic treatment. In addition to aneuploidy’s overall impact on cancer cells, the high prevalence of specific chromosomal changes indicates positive selection and underscores the important role that aneuploidy patterns play in tumor evolution.^65^ However, the genetic mechanisms underlying the selection of these aneuploidy patterns are often unclear.

## 4. What is the genetic basis behind aneuploidy patterns?

Many different theories have been proposed for the mechanisms underlying the frequent aneuploidy observed in cancer. Given the aneuploidy patterns described above, we will focus on theories related to chromosome-specific effects. The prevalence of specific aneuploidies in cancer is not primarily determined by different missegregation rates between chromosomes. Measurements of individual missegregation rates found, at most, a ~ 4-fold difference between chromosomes,^66,67^ which cannot account for the differences in frequencies observed in cancer. Additionally, chromosomes with the highest missegregation rates do not correlate with those with the highest aneuploidy rates in cancer. Larger chromosomes missegregate more frequently, whereas, if anything, the opposite trend is seen in aneuploidy frequency.^35,66,67^ Furthermore, symmetric increases in both chromosome gain and loss would be expected for differences in missegregation rates.

The bias of chromosomes toward being either gained or lost implies that the respective up- or downregulation of specific genes on the aneuploid chromosome drives the observed directional aneuploidy patterns. In agreement with this, measurements of mRNA and protein levels in aneuploid cells show that expression often scales with copy number.^68–71^ However, some degree of dosage compensation does occur for genes on aneuploid chromosomes. In yeast, human cell lines, and tumor cells, ~20–70% of genes display at least partial dosage compensation at the protein level and 5–10% are present at near euploid levels.^68,72,73,74^ Whether an aneuploidy will be selected for is likely a balance between all of the positive and negative effects of the up- or downregulation of hundreds of genes across the chromosome in a particular cellular and environmental context.

### 4.1. Positive selection of aneuploidy patterns

Cancer cells are characterized by their sustained proliferation. Genes that promote proliferation and are often found to be overactivated in cancer are referred to as OGs, while genes that suppress proliferation and are often deactivated in cancer are termed TSGs. Computational analyses of the TCGA have revealed a correlation between the presence of OGs and TSGs on a chromosome and the frequency and directionality (gain vs. loss) of copy number alterations in cancer. OGs are overrepresented on predominantly gained chromosomes and TSGs on lost chromosomes.^75^ In addition, a gain-of-function screen for regulators of proliferation identified cancer type-specific proliferation driver genes present on chromosomes that are frequently gained in a tissue type-specific manner, suggesting that proliferation drivers are cancer type-specific.^76^ Together these studies indicate that the copy number changes of proliferation-associated genes are correlated with, and likely contribute to common and cancer type-specific aneuploidy patterns. Table 1 contains a summary of frequently aneuploid chromosomes and potential proliferation-related driver genes. However, for most of the genes in Table 1, there is no experimental evidence showing that one copy number change is sufficient for conferring a cancer-related phenotype. In addition, dosage compensation of OGs and TSGs on aneuploid chromosomes may complicate the identification of the driver genes. For instance, in cancer cell lines with gain of chromosome 8q, MYC expression was frequently buffered and only showed substantial overexpression upon focal gain.^73^ To date, there are no candidate genes yet identified for many aneuploidy patterns in specific cancer types.

**Table 1.** List of putative driver genes of aneuploidy patterns for the 10 most common pan-cancer chromosome arm aneuploidies. Hallmark OGs and TSGs from the Catalogue of Somatic Mutations in Cancer (COSMIC) database with an aneuploidy frequency above 25% in the indicated tumor type.

Aneuploidy	pan-cancer frequency^13^	putative drivers (COSMIC)	chr. location	Tumor types (frequency >25%, TCGA-tumor type aneuploidy %)^13^
20q gain	31.9%	SRC	20q11.23	colorectal (TCGA-COADREAD 74.4%)
		PTPRT	20q13.11	pan-cancer
		SALL4	20q13.2	pan-cancer
		PTK6	20q13.33	pan-cancer
7p gain	31.0%	EGFR	7p11.2	glioma (TCGA-GBMLGG 48.2%)
		RAC1	7p22.1	pan-cancer
8q gain	29.6%	PREX2	8q13.2	melanoma (TCGA-SKCM 39.6%; TCGA-UVM 56.3%)
		UBR5	8q22.3	colorectal (TCGA-COADREAD 42.7%)
		EIF3E	8q23.1	colorectal (TCGA-COADREAD 42.7%)
		RAD21	8q24.11	pan-cancer (Ewing sarcoma,^50^)
		MYC	8q24.21	pan-cancer (Ewing sarcoma,^50^)
1q gain	27.8%	DDR2	1q23.3	squamous cell carcinoma (PANSCC 31%)
		MDM4^11^	1q32.1	breast (TCGA-BRCA 62.3%)
7q gain	27.3%	CUX1	7q22.1	pan-cancer
		MET	7q31.2	papillary renal (TCGA-KIRP 60.8%)
		BRAF	7q34	pan-cancer
20p gain	26.4%	-		
17p loss	24.6%	MAP2K4	17p12	breast (TCGA-BRCA 39.3%), colorectal (TCGA-COADREAD 43.8%)
		NCOR1	17p12	breast (TCGA-BRCA 39.3%)
		TP53	17p13.1	pan-cancer
5p gain	24.1%	DROSHA	5p13.3	bladder carcinoma (TCGA-BLCA 34.4%)
		TERT	5p15.33	pan-cancer
9p loss	21.8%	CDKN2A	9p21.3	pan-cancer
22 loss	20.5%	EWSR1	22q12.2	mesothelioma (TCGA-MESO 66.7%)
		EP300	22q13.2	colorectal (TCGA-COADREAD 29.9%), breast (TCGA-BRCA 34.3%)

Recent evidence highlights the context-dependent nature of aneuploidy formation. An analysis of the lineage-specific recurrence of partial chromosome gains and losses shows that cancer type-specific driver genes underly the selection of pan-cancer aneuploidy patterns. For instance, monosomy of chromosome arm 3p displays different regions driving positive selection across tumor types.^13^ These data support the possibility that multiple different cancer type-specific drivers underly selection, even for pan-cancer aneuploidy patterns.

Although direct evidence for specific genes contributing to the selection of aneuploidies in cancer is rare, there are many examples in model systems. The first observation of this phenomenon was in the pathogenic yeast *Candida albicans*, where drug resistance was conferred by the gain of extra copies of the left arm of chromosome 5, containing the resistance genes *ERG11* and *TAC1*.^77,78^ Additionally, many examples of obtaining resistance to stress conditions through specific aneuploidy chromosomes have been identified in the nonpathogenic yeast *Saccharomyces cerevisiae* (reviewed in ^79^). For example, adaptation of haploid yeast cells to mutations that induce high rates of chromosome missegregation led specifically to the frequent gain of 5 different chromosomes in the adapted cells. Relocating one gene, *SLI15*^*INCENP*^, from its endogenous chromosome to a different chromosome was sufficient to alter the aneuploidy pattern of adapted cells toward gaining the alternate chromosome.^80^ In mice, clonal selection of lymphomas led to the recurrent acquisition of chromosome 15 gain, which contains the oncogene *Myc*. Expression of human *MYC* on chromosome 6 resulted in the additional selection of this chromosome in lymphomas.^44^ For the chromosome 9p monosomy that underlies defects in anti-tumor immunity, a mouse model of pancreatic cancer was used to identify the loss of type 1 interferon genes contributing to the phenotype.^81^ These and other studies in model organisms demonstrate that the expression changes of specific genes contribute to the selection of aneuploid chromosomes.

Recently, progress has been made in identifying specific genes that at least partially contribute to aneuploidy-dependent advantages in certain cancer cell lines. In one study, the authors restored trisomic chromosome arms to diploid copy number levels in human cancer cell lines, and could subsequently observe reduced growth both in culture and in mouse xenographs.^11^ The p53 negative regulator *MDM4* was identified as a contributor to the growth advantage of chromosome 1q gain. Interestingly, the authors also found a negative correlation between 1q gain and p53 mutation, suggesting that 1q gain could potentially act as an alternative pathway for p53 inactivation. However, the elimination of other common aneuploidies displayed little-to-no changes in phenotype, highlighting the difficulties in determining the basis for aneuploid chromosome selection.^11^ Another study investigating the selection of 1q gain in breast cancer identified increased Notch-signaling as a driver underlying the gain of the chromosome arm. In both mammary epithelial cell lines and mammary tumors with 1q gain, they found increased expression of the γ-secretase genes APH1A, NCSTN, and *PSEN2*. Expression levels of *NCSTN*, present on 1q, positively correlated with Notch activation.^82^ Together, these two studies suggest that 1q could be gained for multiple independent reasons. The proliferative advantage of chromosome 8 trisomy in the context of the oncogenic *EWS-FLI1* fusion gene could be partially attributed to the copy number gain of the gene for the cohesin subunit *RAD21*. Mild *RAD21* overexpression partially relieves the replication stress caused by the oncogenic fusion protein.^50^ Recent studies have also shed light on the genetic link between trisomy 21 and hematopoietic cancers, pointing toward the dysregulation of *RUNX1* and likely other genes on chromosome 21 contribute to the early stages of hematopoiesis.^83,84^

Overall, there is substantial evidence that expression changes of particular genes can act as the positive selective forces for evolving through aneuploidy. However, in the majority of cancer-associated aneuploidies, it is not clear which genes are the source of the selection, nor if the same genes are providing the selective force in different cancer types.

### 4.2. Negative selection of aneuploidy patterns

Negative selection of gene mutations is generally believed to be a negligible force on tumor growth, as the majority of mutations in the cell will be silent or suppressed by the homologous allele.^85,86^ By contrast, aneuploidies affect hundreds of genes all at the same time, which can substantially impact the fitness of the cells. The genetic imbalance of somatic aneuploidy leads to a reduced fitness in every species studied.^37,69,87^ Furthermore, there are strong negative correlations between fitness and the number of genes on aneuploid chromosomes,^87,88,89^ indicating that the imbalanced expression of genes underlies the growth defects. Besides defective growth, aneuploidy is associated with genomic instability, replication stress, proteotoxic stress, DNA damage and apoptosis.^90,91,92,93,94^ This is consistent with the observation that aneuploidy is very rare in healthy tissue.^95^ Additionally, only three autosomal germline aneuploidies are compatable with live births, the gains of chromosome 13, 18 and 21. These three chromosomes have the fewest number of genes. All other autosomal aneuploidies lead to early spontaneous abortion.^96^

In some studies, the correlation between chromosome gene number and negative selection is also seen in cancer. For ovarian carcinoma, the frequency of losing or gaining a chromosome arm negatively correlates with the number of genes on the chromosome arm.^97^ In solid tumors, the number of genes on chromosomes inversely correlates with the percentage of chromosomes being lost, indicating that the loss of gene-rich chromosomes is under negative selection pressure.^35^ These observations support the hypothesis that aneuploidies are negatively or positively selected based on the balance between beneficial and detrimental consequences of the induced gene copy number alterations.

These negative selection forces from gene expression changes could either be general effects on cellular fitness or specifically inhibit cancer progression. Genes that are negatively selected due to decreased fitness would be either haploinsufficient for chromosome loss or triplosensitive for chromosome gain. Intriguingly, a study in yeast found that haploinsufficient genes are frequently triplosensitive as well, suggesting that certain genes have an narrow range of optimal expression.^98^ Although haploinsufficiency and triplosensitivity of individual genes rarely lead to strong phenotypes at the cellular level, the additive effects of many such genes across an entire chromosome likely lead to the observed growth defects.^99^ In humans, an extensive meta-analysis of rare copy number variations from over 950.000 individuals spanning 54 disease phenotypes identified 3,635 dosage sensitive protein-coding genes.^100^

As mentioned above, there is a correlation between OGs on a chromosome and how frequently it is gained and TSGs and how frequently it is lost.^75^ Intriguingly, the reverse correlations also exist. OGs negatively correlate with chromosome loss and TSGs negatively correlate with chromosome gain. This points toward cancer-specific sources of negative selection. This could result from cancer progression being inhibited by the overexpression of TSGs on chromosome gains and the decreased expression of OGs on chromosome losses. For some tumor suppressor genes, overexpression reduces proliferation, transformation, and or malignancy. Overexpression of the TSG *PTEN* has been shown to reduce the amount of tumor growth in a mouse mammary cancer model of *Wnt-1* overexpression.^101^ Similarly, overexpression of the TSG *RB1* inhibits tumor progression in metastatic melanoma cells.^102^ Since many proto-oncogenes are involved in promoting cell cycle entry, they would therefore potentially decrease proliferation when decreased in expression.

As mentioned above, engineered aneuploid chromosomes in human cancer cells revert to euploidy following mouse xenografts, suggesting that these negative selective pressures occur in cancer as well. Recent work from Shih and colleagues in which they analyzed the length distribution of large partial arm somatic copy number alterations (SCNAs) from the TCGA provides evidence for the existence of specific regions of aneuploid chromosomes under negative selection in cancer. Out of 193 loci identified as being under selection, 103 were categorized to be under negative selection.^13^ Unlike with other types of genomic alterations, there are substantial data demonstrating negative selection of aneuploid chromosomes. It is therefore surprising that aneuploidy is so common in cancer, and the forces of positive selection must be especially strong to overcome the negative selection.

### 4.3. Selection forces underlying aneuploidy patterns that can be either positive or negative

Many types of changes to the genome can either promote or inhibit growth depending on the context. For example, multiple aneuploid chromosomes within the same cell have the potential to affect each other in complex ways. Evidence for this comes from positive and negative correlations between specific aneuploid chromosomes in cancers.^49,80,103^ Such correlations have also been observed in cellular adaptation assays. For instance, chronic myeloid leukemia cells adapted to the drug reversine through the acquisition of many frequent aneuploidies; however, the gain of chromosome 7 strongly negatively correlated with the loss of chromosome 14. Over time, chromosome 14 loss became much more frequent as chromosome 7 gain was lost from the cell populations. These results suggest negative genetic interactions between aneuploid chromosomes.^8^ Direct evidence for such interactions between specific aneuploid chromosomes comes from experiments using budding yeast. By examining the karyotypes of yeast after adaptation to the high rates of chromosome missegregation, we previously identified specific positive and negative correlation between aneuploid chromosomes. Engineered yeast strains with chromosome gain combinations showed growth rates that differed from what was predicted by the growth of each individual aneuploid chromosome on its own.^80^ In summary, genetic interactions between aneuploid chromosomes provides a plausible mechanism for positive as well as negative selection of aneuploidy patterns in cancer.

Loss of heterozygosity (LOH) occurs when a heterozygous genotype becomes homozygous through the loss or replacement of one of the two copies. In the case of aneuploidy, loss of one of the two copies of a chromosome eliminates all heterozygosity across its length. In addition to copy-number loss LOH, the chromosome can be subsequently duplicated, resulting in copy-number-neutral LOH or even amplified resulting in gain-of-copy LOH.^104,105^ A recent study suggests that across the 33 TCGA tumor types an average of 16% of genes display LOH, mostly from copy-number-loss LOH. This high frequency of LOH suggests a relevant mechanism in cancer evolution for positively selecting chromosome loss.^105^ LOH of chromosome arms 18p, 17p, 10p, 4p, 18q, 9p and 8p is frequent in cancer and TSGs are enriched in LOH regions.^104^ Conversely, LOH can be under negative selection. The most extreme example of this would be for recessive loss-of-function alleles of essential genes that are uncovered by chromosome loss. The negative effects of LOH on specific genes could also be potential targets for cancer therapy.^105^ One study determined the presence of negative selection copy number loss of genomic regions where harmful mutations cannot be balanced out by healthy allele copies.^106^

### 4.4. Neutral selection of aneuploidy patterns

Neutral selection is thought to be acting on most of the acquired gene mutations in cancer, which are referred to as passenger mutations.^86^ However, neutral selection for aneuploidy would seem unlikely due to the expression changes of hundreds of genes at once as described above. However, one candidate for neutral selection shaping aneuploidy patterns could be the loss of the sex chromosomes in specific cancer types. In the TCGA, 1504 of 5014 analyzed male tumors (30%) exhibited complete or relative loss of Y (LOY), ranging from 80% in kidney renal papillary cell carcinoma (KIRP) to 1.3% in Pheochromocytoma and Paraganglioma.^107^ In most analyzed cancer types LOY correlated with aneuploidy rates as a whole, suggesting neutral selection. However, at least three tumor types – KIRP, uveal melanoma (UVM, 47% LOY) and kidney renal cell carcinoma (KIRC, 42% LOY) – have especially high frequencies of LOY, suggesting positive selection.^107^ Additionally, loss of the Y chromosome in bladder cancer has recently been demonstrated to drive evasion of the adaptive immunity.^108^ Therefore, recurrent aneuploidy of even the smallest chromosome likely has a role in tumor development. The loss of the X chromosome (LOX) is identified in 757 of 5394 (14%) analyzed female tumors in the TCGA.^107^ In female cells, one of the two X chromosomes is silenced, suggesting minimal impact from the loss of the silenced copy. However, not much is known about the role of X chromosome in tumorigenesis. Overall, with some potential exceptions regarding the sex chromosomes in specific cancer types, passenger aneuploidies with neither negative nor positive effects on cancer fitness are likely rare.

**Figure 3.**
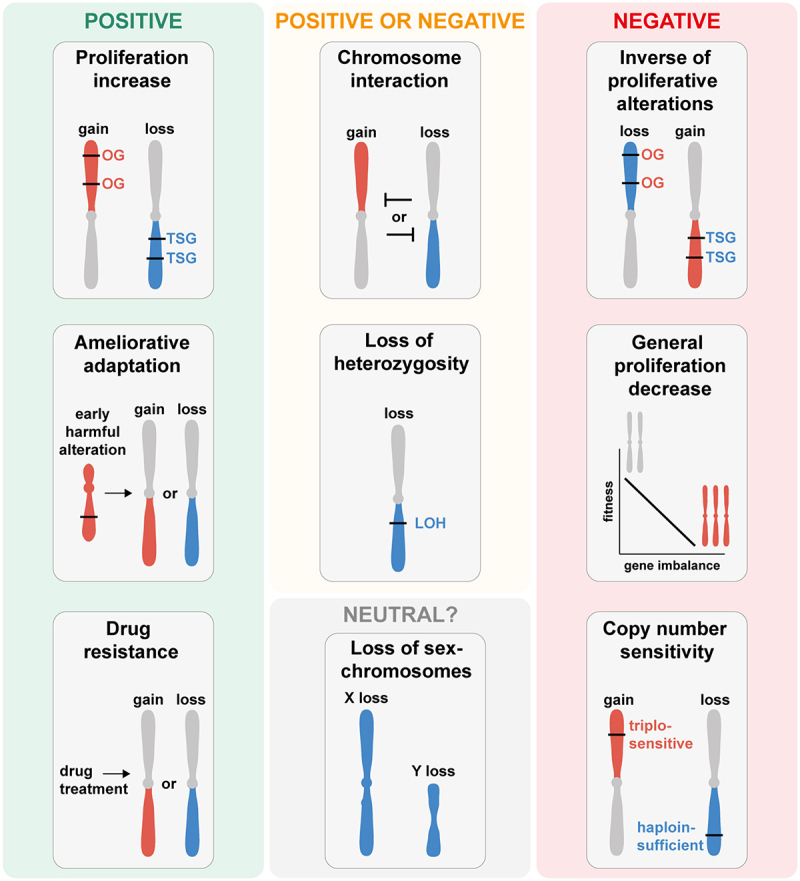
Selection forces underlying the formation of aneuploidy patterns in cancer and human cell lines. Illustration of selection forces that shape the formation of aneuploidy patterns based on the observations from cancers, human cell lines, and model systems. Depending on environmental factors, cell type, and chromosome identity, aneuploidy patterns can be driven by positive, negative, or neutral selection forces. Positive selection forces underlying the copy number changes of a chromosome arm include: drivers of increased proliferation (upper left panel), compensating for harmful alterations (middle left panel) and conferring drug resistance (lower left panel). Selection forces that could be positive or negative depending on the context include: copy number loss resulting in loss of hetero-zygosity (LOH) and genetic interactions between aneuploid chromosomes (upper middle panels). Negative selection forces against chromosome copy number changes include: antagonization of proliferative alterations (upper right panel), the general proliferation decrease resulting from the imbalanced expression of many genes (middle right panel), and triplosensitive or haploinsufficient genes (lower right panel). Neutral selection of aneuploidy patterns is likely very rare and might only occur for aneuploidy of the sex chromosomes in specific cancer or cell types (lower middle panel). Chromosome arm gain is depicted in red, chromosome arm loss in blue.

In sum, there are likely many positive and negative selection forces acting simultaneously for any individual aneuploid chromosome (Figure 3). Whether an aneuploid chromosome is beneficial or not for tumor growth depends on the cumulation of these forces. Depending on the context influencing the selection forces, aneuploidies that can be beneficial in a certain tumor type or cell line can be disadvantageous in another. This becomes even more complex in the context of additional aneuploid chromosomes, mutations, and external selective forces.

## 5. How many genes drive aneuploidy patterns?

One of the key questions remaining for the selection of aneuploid patterns relates to how many genes are involved in contributing to their selection. Are these chromosomes being gained or lost due primarily to a single gene or the combinatorial impact of expression changes of many genes? This question gets to the heart of why aneuploidy may be so frequently observed in cancer as compared to other forms of mutation. There is some intriguing correlative evidence suggesting multiple genes may contribute to aneuploid chromosome selection. For example, the four most frequently mutated tumor suppressor genes in ccRCC (*VHL*, *PBRM1*, *SETD2* and *BAP1*) are all located on chromosome arm 3p, and 3p loss is the most common aneuploidy in this cancer type.^109^ However, determining the number of genes that contribute to the formation of aneuploidy patterns has been challenging and there is currently a dearth of direct evidence for any particular genes being necessary or sufficient.

One source of examples of aneuploidy patterns comes from *in vitro* evolution of model systems, where specific genes have been implicated in aneuploid chromosome selection to a variety of stresses and mutations. In some cases, these genes have been deleted from the aneuploid chromosomes to determine the degree to which they contribute to the observed phenotypes. The model system where such dependencies have been primarily studied is budding yeast. There have been a few cases where a single gene has been found to be necessary and sufficient to fully recapitulate an aneuploidy-associated phenotype.^80,110^ However, the majority of cases have been shown to involve at least two genes.^79^ Using a mouse model with a partial chromosome deletion syntenic to human chromosome 17p13.1 encompassing the *TP53* gene, Liu and colleagues showed that the advantageous effect resulting from the deletion of human chromosome 17p on cancer development is a consequence of both the absence of *TP53* and the reduced levels of linked tumor suppressor genes working in conjunction, i.e. *Eif5a* and *Alox15b*.^111^

There have been a few cases where this question has been directly addressed in human cells. In a model for Ewing sarcoma that leads to the frequent gain of chromosome 8, extra copies of the genes for *MYC* and the cohesin subunit *RAD21* were shown to contribute to an increase in cell proliferation when combined. However, together they were still not sufficient to fully recapitulate the aneuploid phenotype, suggesting that additional genes on chromosome 8 contribute to the phenotype.^50^ In a recent study of cancer cell lines with 1q gain, it was shown that deletion of the extra copy of *MDM4* gene reduced proliferation, but not to the same extent as removing the extra copy of the entire arm.^11^ In human liver cancer cell lines, the loss of chromosome arm 8p leads to increased migration and invasion. The overexpression of individual candidate genes located on chromosome 8p within the context of chromosome 8p deletion only partially reversed the migratory phenotype. However, the simultaneous overexpression of a combination of four genes led to a migratory capacity comparable to their overexpression in euploid wildtype cells.^12^ These findings suggest that multiple genes contribute to the selection of aneuploid chromosomes in cancer.

One possibility for how to identify if multiple genes drive a phenotype of an aneuploidy is through engineering segmental SCNAs. This has been done for the chromosome arm deletions of 6p and 13q to determine regions responsible for drug resistance.^8^ This technique could be used to uncover regions on other aneuploid chromosomes that drive aneuploidy phenotypes, helping to exactly determine how many chromosomal regions contribute to aneuploid chromosome selection. For now, the identification of the number of genes typically involved in aneuploid chromosome selection and the extent to which they act additively or synergistically remains to be determined.

## 6. Influence of whole genome duplication on aneuploidy patterns

After aneuploidy, whole genome duplication (WGD) is the second most common chromosome-level genome alteration in cancer cells. WGD occurs in approximately one third of human tumors^112,113,114^ and is associated with worse prognosis and survival.^112^ Generally, WGD is correlated with a higher degree of aneuploidy.^6,112^ Most WGD tumors are near triploid which indicates that WGD might be more permissive for chromosome loss.^112,115^ Whether chromosome losses occur before or after WGD seems to differ between cancer types and type of analyses. For instance, researchers have identified that 70% of acquired heterozygous losses arose after WGD.^112^ In contrast, Carter and colleagues identified that chromosome arm copy number changes occur mostly prior to genome doubling.^116^ This is also seen in basal-like breast carcinoma, in which WGD occurred after multiple chromosome losses.^117^ Additionally, WGD tumors have a more diverse spectrum of aneuploidies indicating greater tolerance to various chromosomes being aneuploid.^113^ The better tolerance might be partially explained by the smaller relative effect of one copy number change in tetraploid cells compared to diploid cells. The higher ploidy decreases the impact of expression imbalances from gaining one chromosome copy from 1/3 to 1/5 and losing one copy from 1/2 to 1/4. Associated with reduced effect of chromosome loss, polyploidy was proposed to protect from LOH.^118^ A recent study suggests that WGD influences complex aneuploidy patterns in tumors. In yeast and in human tumors, complex aneuploidy patterns are affected by initial ploidy.^80,113^ In cancer, the frequent co-occurrence of certain chromosome arms can change to mutual exclusivity and vice versa depending on the WGD status.^113^ However, in general, tumor type-specific aneuploidy patterns are independent of WGD status.^113^ Collectively, these analyses suggest minimal influence of WGD on pan-cancer and cancer type-specific aneuploidy patterns but identified a role of WGD in the formation of complex aneuploidy patterns.

## 7. Relevance of aneuploidy patterns for cancer treatment

The absence or presence of aneuploidy in cells has been utilized as a fundamental differentiator between transformed and healthy cellular states in various tumor types, rendering aneuploidy an attractive and selectively targetable hallmark of tumors.^119^ This selective targeting approach holds promise for therapies that can exert their effects predominantly on cancer cells while minimizing adverse impacts on healthy counterparts.

One approach being explored is targeting the specific vulnerabilities associated with aneuploid chromosomes in general, such as proteotoxic stress,^120,121^ DNA damage response,^122^ or sphingolipid synthesis.^123^ In addition, therapies based on specific frequent aneuploidies may hold promise as personalized therapeutic strategies. One potential benefit of targeting the specific aneuploidy patterns of a given tumor would be greater specificity for elimination of the specific tumor cells compared to general chemotherapeutics. This approach seems especially encouraging and supported by the recent identification that cancer cell lines are “addicted” to their aneuploidies.^11^

However, there are currently many challenges associated with developing treatments targeting aneuploid chromosomes. Firstly, we do not know the genetic basis that drives the selection of most aneuploid chromosomes in tumors, making it impossible to design specific strategies for treatments based on their specific contributions to cancer. Second, we know little about which of the non-driver genes on the aneuploid chromosome might underlie the chromosome-specific negative proliferation effects. These other dosage sensitive genes could be targeted with treatments exploiting chromosome-specific vulnerabilities.^124^ Recently, Girish and colleagues discovered that the frequent gain of chromosome 1q in breast cancer also changes the protein abundance of UCK2 making cells with a chromosome 1q gain more sensitive to certain drugs.^11^ Gene imbalances from aneuploidy could also be exploited for synthetic lethal interactions. A recent study identified deletion of the reactive oxygen sensing enzyme NUDT17 as synthetic lethal with an engineered chromosome 8p deletion cell line.^12^ Finally, one limitation for targeting aneuploidy patterns instead of gene mutations could be the relatively easy reversal of the targeted aneuploid chromosome to euploid levels in cancer cells. Changing the copy number of one chromosome can be achieved through a mis-segregation event during cell division which is very likely to occur in cancer cells. In chromosomally unstable colorectal cancer cell lines, it has been observed that in one out of five cell divisions, chromosomes become mis-segregated.^125–127,^ By comparison, reverting a gene mutation will occur much less frequently. Therefore, targeting aneuploidy patterns in tumors might only be realistic in the context of combinatorial treatments that additionally target other vulnerabilities.

## 8. Concluding remarks and future perspectives

In summary, the high prevalence and specificity of different types of aneuploidy patterns underscores their context-dependent relevance for tumorigenesis and indicates positive selection. There is growing evidence for aneuploidy patterns playing cancer type-specific roles in tumor initiation, progression, metastasis formation, immune evasion and resistance to therapeutic treatment. Despite being pervasive in human tumors, the genetic basis of aneuploidy pattern selection largely remains to be uncovered. The selection of aneuploidies likely depends on various positive and negative forces which amount to either a net beneficial or detrimental consequence on tumor cell growth. While there is some evidence of different driver genes shaping aneuploidy patterns in cancer, further research is necessary to identify the most impactful driver genes. In addition, cancer type-specific genetic vulnerabilities associated with certain aneuploidy patterns are just beginning to be investigated.

## Abbreviations

BLCA: **BL**adder Urothelial **CA**rcinoma
BRCA: **BR**east Invasive **CA**rcinoma
ccRCC: **c**lear **c**ell **R**enal **C**ell **C**arcinoma
CIN: **C**hromosome **IN**stability
COAD: **CO**lon **AD**enocarcinoma
COADREAD: **CO**lon **AD**enocarcinoma (**COAD)** and **RE**ctum **AD**enocarcinoma (**READ**)
COSMIC: **C**atalogue **O**f **S**omatic **M**utations **I**n **C**ancer
FHWT: **F**avorable **H**istology **W**ilms **T**umor
GBM: **G**lio**B**lastoma **M**ultiforme
GBMLGG: **G**lio**B**lastoma **M**ultiforme and Brain **L**ower **G**rade **G**lioma
HCC: **H**epato**C**ellular **C**arcinoma
HNC: **H**ead and **N**eck **C**ancer
KIRP: **KI**dney **R**enal **P**apillary Cell Carcinoma
LOH: **L**oss **O**f **H**eterozygosity
LOX: **L**oss **O**f **X** chromosome
LOY: **L**oss **O**f **Y** chromosome
MESO: **MESO**thelioma
NSCLC: **N**on-**S**mall **C**ell **L**ung **C**ancer
OG: **O**nco**G**ene
PANSCC: **PAN-S**quamous **C**ell **C**arcinoma
SCNAs: **S**omatic **C**opy **N**umber **A**lterations
SKCM: **SK**in **C**utaneous **M**elanoma
SNV: **S**ingle **N**ucleotide **V**ariants
TCGA: **T**he **C**ancer **G**enome **A**tlas
TSG: **T**umor **S**uppressor **G**ene
UVM: **UV**eal **M**elanoma
WGD: **W**hole **G**enome **D**uplication
